# A data-driven ultrasound approach discriminates pathological high grade prostate cancer

**DOI:** 10.1038/s41598-022-04951-3

**Published:** 2022-01-17

**Authors:** Jun Akatsuka, Yasushi Numata, Hiromu Morikawa, Tetsuro Sekine, Shigenori Kayama, Hikaru Mikami, Masato Yanagi, Yuki Endo, Hayato Takeda, Yuka Toyama, Ruri Yamaguchi, Go Kimura, Yukihiro Kondo, Yoichiro Yamamoto

**Affiliations:** 1 Department of Urology, Nippon Medical School Hospital, Tokyo, 113-8603 Japan; 2 Pathology Informatics Team, RIKEN Center for Advanced Intelligence Project, Tokyo, 103-0027 Japan; 3 Department of Radiology, Nippon Medical School Hospital, Tokyo, 113-8603 Japan

**Keywords:** Prostate, Computational science

## Abstract

Accurate prostate cancer screening is imperative for reducing the risk of cancer death. Ultrasound imaging, although easy, tends to have low resolution and high inter-observer variability. Here, we show that our integrated machine learning approach enabled the detection of pathological high-grade cancer by the ultrasound procedure. Our study included 772 consecutive patients and 2899 prostate ultrasound images obtained at the Nippon Medical School Hospital. We applied machine learning analyses using ultrasound imaging data and clinical data to detect high-grade prostate cancer. The area under the curve (AUC) using clinical data was 0.691. On the other hand, the AUC when using clinical data and ultrasound imaging data was 0.835 (*p* = 0.007). Our data-driven ultrasound approach offers an efficient tool to triage patients with high-grade prostate cancers and expands the possibility of ultrasound imaging for the prostate cancer detection pathway.

## Introduction

Prostate cancer is one of the most commonly diagnosed cancers in elderly men^1^. Ultrasound imaging is widely used in prostate cancer screening because it is nonionizing, low-cost, and safe. However, its low resolution and high inter-observer variability deteriorate the accuracy of ultrasound diagnosis^2^. Prostate cancer is a heterogeneous disease that ranges from indolent to aggressive^3^. Pathological grading is considered an important factor in predicting the prognosis of prostate cancer patients^4^. High-grade cancer tends to metastasize and is usually considered a castration-resistant prostate cancer^5^. On the other hand, older men with low-grade cancer who undergo treatment may experience complications without reducing their risk of dying from prostate cancer^6^. Accurate diagnosis through prostate cancer screening enables optimization of cancer management. Since pathological cancer grading determines therapeutic strategies, it is necessary not only to detect the cancer, but also to estimate the pathological grading, such as the Gleason score^7^, which would be desirable to know even during ultrasound examination.

Artificial intelligence (AI) technologies, including deep learning algorithms, are gaining extensive attention due to their excellent performance in image classification and object detection. Recently, these algorithms have been useful tools in the analysis of medical images of various cancers, such as breast cancers^8^, brain tumors^9^, lung cancers^10^, esophageal cancers^11^, skin malignancies^12^, and prostate cancers^13–15^. The robust performance of the deep learning algorithm indicates its potential clinical use for screening on computed tomography (CT) images of patients with suspected coronavirus disease 2019 (COVID-19) pneumonia^16,17^. Furthermore, deep learning techniques have also been applied to ultrasound imaging. Li et al. reported that their deep convolutional neural network model showed similar sensitivity in identifying thyroid cancer compared with skilled radiologists^18^. Gu et al. proposed an automated three-dimensional segmentation approach using deep learning on ultrasound images for breast cancer^19^. In the field of prostate ultrasound image analysis, deep learning techniques enable accurate automatic segmentation^20^. Furthermore, several studies proposed a deep learning framework to detect prostate cancer using contrast-enhanced ultrasound images^21,22^. However, it is highly challenging to estimate pathology-level cancer grading using deep learning based on ordinary ultrasound images. In this study, we aim to estimate pathological high-grade cancer using ordinary ultrasound images and limited clinical data.

## Results

### Image and patient characteristics

**Figure 1:**
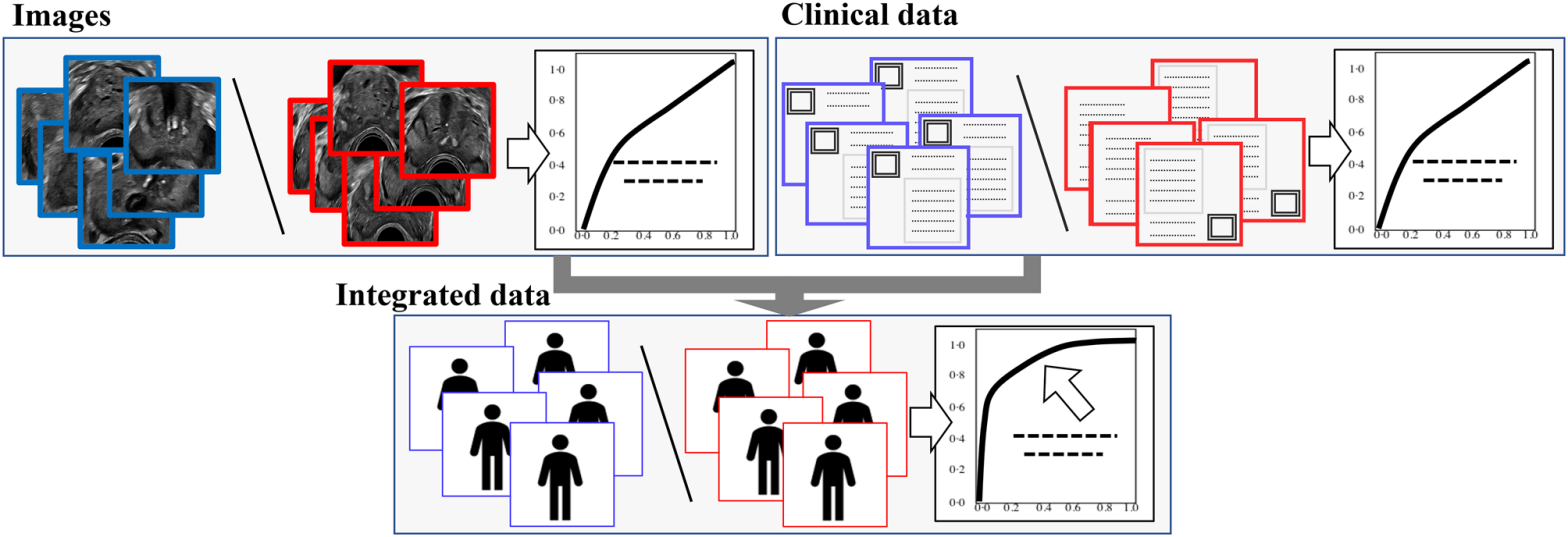
Flowchart of our study. Deep learning analysis for ultrasound images (upper left images), clinical data (upper right images), and integrated data (lower images).

A flowchart of the study is presented in Fig. 1. We evaluated prediction accuracies for prostate cancer using the following data sets: still ultrasound image data (left upper box), clinical data (age and prostate-specific antigen [PSA]) (right upper box), and integrated data (ultrasound image data, total prostate volume [TPV] derived from ultrasound images, PSA density [PSAD], age, and PSA) (lower box).

Table 1 shows 691 cases (2676 images) that underwent ultrasound-guided prostate biopsy (systematic biopsy and magnetic resonance imaging [MRI]-targeted biopsy) at the Nippon Medical School Hospital. The median age (interquartile range) of all cases was 71 (65–76) years, and that of the cancer cases was 72 (66–77) years. Patients with cancer were significantly older than those without cancer (*p* < 0.001). The median PSA value of all the cases was 8.3 (5.8–14.0) ng/mL, and that of the cancer cases was 9.5 (6.7–20.5) ng/mL. The PSA level was significantly higher in cancer cases than in non-cancer cases (*p* = 0.002). The median TPV of all the cases was 35.0 (25.8–50.6) cm^3^, and that of the cancer cases was 30.9 (23.0–42.6) cm^3^. TPV was significantly lower in cancer cases than in non-cancer cases (*p* < 0.001). The median PSAD of all cases was 0.245 (0.138–0.482) ng/mL/cm^3^, and that of the cancer cases was 0.352 (0.207–0.681) ng/mL/cm^3^. PSAD was significantly higher in cancer cases than in non-cancer cases (*p* < 0.001). The number of biopsy Gleason scores was 6 (47 cases), 7 (215 cases), 8 (79 cases), 9 (94 cases), and 10 (1 case). High-grade cancer (Gleason score ≥ 8) accounted for 39.9% of the cancer cases.

**Table 1:** Patient characteristics.

	Total	Cancer cases	Non-cancer cases	*p* value
Cases, n (images, n)	691 (2676)	436 (1691)	255 (985)	–
**Age (years)**				
Median (IQR) Mean (SD)	71, 65–76 69.9 ± 8.59	72, 66–77 71.3 ± 8.18	69, 62–74 67.4 ± 8.71	< 0.001
**PSA (ng/mL)**				
Median (IQR) Mean (SD)	8.3, 5.8–14.0 128.9 ± 1034.7	9.5, 6.7–20.5 198.9 ± 1297.5	6.6, 4.9–10.4 9.20 ± 10.38	0.002
**TPV (cm**^**3**^**)**				
Median (IQR) Mean (SD)	35.0, 25.8–50.6 42.8 ± 29.1	30.9, 23.0–42.6 37.1 ± 27.4	45.9, 32.5–62.0 52.5 ± 29.2	< 0.001
**PSAD (ng/mL/cm**^**3**^**)**				
Median (IQR) Mean (SD)	0.245, 0.138–0.482 2.85 ± 18.1	0.352, 0.207–0.681 4.39 ± 22.6	0.140, 0.0939–0.214 0.201 ± 0.288	< 0.001
**Gleason score, n**				
6	47	47	–	–
7	215	215		
8	79	79		
9	94	94		
10	1	1		

*PSA* prostate-specific antigen, *TPV* total prostate volume, *PSAD* PSA density, *IQR* interquartile range, *n* number, *SD* standard deviation.

### Classification of prostate cancers (n = 691)

#### Image-level classification using a deep neural network

We performed two sets of deep learning analyses using different labels. First, we applied a deep neural network, Xception^23^, to ultrasound image data with labels of the cancer group (positive, Gleason score ≥ 6) and the non-cancer group (negative). The area under the curve (AUC) of the cancer classification was 0.693 (95% confidence interval [CI] 0.640–0.746) (Table 2). Next, we applied the deep neural network to the same ultrasound image data with labels of the high-grade cancer group (positive, Gleason score ≥ 8) and the others (negative). The AUC of the high-grade cancer classification was 0.723 (95% CI 0.659–0.788) (Table 2). Note that we only used ultrasound images for these classifications. Supplementary Fig. S1 shows the receiver operating characteristic (ROC) curves for the classification accuracy.

**Table 2:** AUCs of the cancer grading classification (n = 691).

	Images	Clinical data	Image integration	Data integration	*p* value*
Cancer classification	Deep learning 0.693 (95% CI 0.640–0.746)	Ridge 0.671 (95% CI 0.563–0.779)	Deep learning + ridge 0.774 (95% CI 0.680–0.868)	Deep learning + ridge 0.789 (95% CI 0.697–0.880)	0.104
		Lasso 0.671 (95% CI 0.562–0.779)	Deep learning + Lasso 0.774 (95% CI 0.680–0.868)	Deep learning + Lasso 0.779 (95% CI 0.686–0.873)	0.141
		**SVM** **0.702 (95% CI 0.598–0.806)**	**Deep learning + SVM** **0.776 (95% CI 0.683–0.870)**	**Deep learning + SVM** **0.807 (95% CI 0.719–0.894)**	0.051
High-grade cancer classification	Deep learning 0.723 (95% CI 0.659–0.788)	Ridge 0.675 (95% CI 0.564–0.786)	Deep learning + Ridge 0.816 (95% CI 0.725–0.908)	Deep learning + Ridge 0.822 (95% CI 0.736–0.908)	0.012
		Lasso 0.665 (95% CI 0.553–0.777)	Deep learning + Lasso 0.816 (95% CI 0.724–0.907)	Deep learning + Lasso 0.824 (95% CI 0.737–0.911)	0.009
		**SVM** **0.691 (95% CI 0.582–0.801)**	Deep learning + SVM 0.816 (95% CI 0.725–0.908)	**Deep learning + SVM** **0.835 (95% CI 0.753–0.916)**	0.007

The bold text indicates the highest value of AUCs.

*AUC* area under the curve, *CI* confidence interval, *SVM* support vector machine.

*AUC of clinical data versus that of data integration.

#### Case-level classification based on clinical data

We also applied logistic regression and support vector machine (SVM)^24,25^ analyses to the clinical data with the aforementioned labels: (1) cancer classification label: cancer (Gleason score ≥ 6) or non-cancer group, (2) high-grade cancer classification label: high-grade cancer group (Gleason score ≥ 8) or the other. Age and PSA were used as clinical data in this study because these clinical data are known to be important factors in the screening of prostate cancers^26^. Table 2 presents the AUCs for each classification. The AUC of the cancer classification was 0.702 (95% CI 0.598–0.806), and that of the high-grade cancer classification was 0.691 (95% CI 0.582–0.801) (SVM). Note that we used only clinical data for this classification. Supplementary Fig. S2 shows the ROC curves for these classifications.

#### Case-level classification using an image integration approach

Next, we applied logistic regression and SVM to three ultrasound image data pre-analyzed by deep learning. We selected the ultrasound images with the top three highest probabilities (|P_dl_ − 0.5|, P_dl_: predicted probability of the deep learning classification) in every case. Table 2 shows the AUCs. Supplementary Fig. S3 shows the ROC curves for these classifications. The highest value of AUCs in cancer classification was 0.776 (95% CI 0.683–0.870) (SVM), and that in the high-grade cancer classification was 0.816 (95% CI 0.725–0.908).

#### Case-level classification using a data integration approach

**Figure 2:**
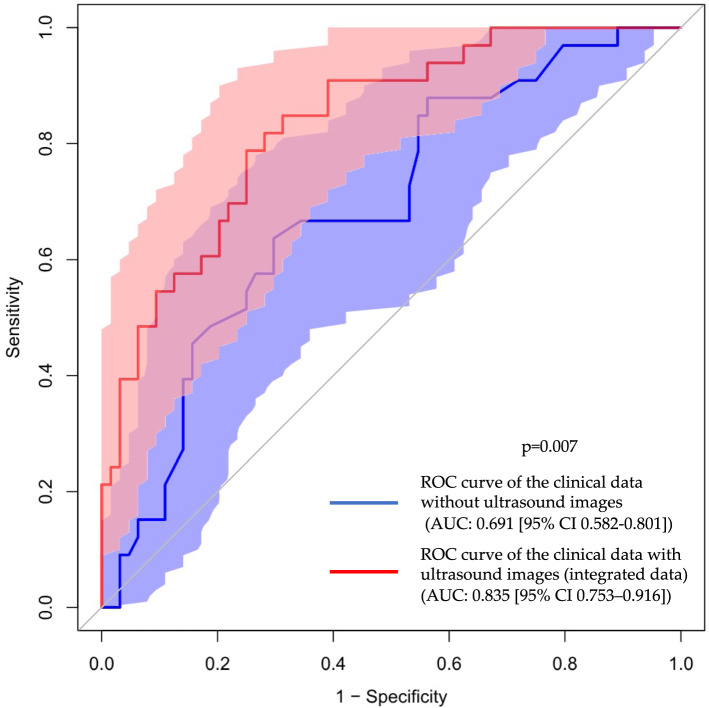
ROC curves of high-grade cancer classification (n = 691: systematic biopsy and MRI-targeted biopsy cases). Blue line: ROC curve of the clinical data without ultrasound images. Red line: ROC curve of clinical data with ultrasound images (integrated data). Light blue area: 95% CI for the ROC curve of the clinical data without ultrasound images. Light red area: 95% CI for ROC curve of clinical data with ultrasound images (integrated data). *ROC* receiver operating characteristic, *AUC* area under the curve, *CI* confidence interval.

Finally, we applied logistic regression and SVM analyses to the integrated data (three ultrasound image data pre-analyzed by deep learning, TPV, PSAD, age, and PSA). We also selected the ultrasound images with the top three highest probabilities in every case. Table 2 shows the AUCs. Supplementary Fig. S4 shows the ROC curves for these classifications. In the integrated data, the highest value of AUCs in cancer classification was 0.807 (95% CI 0.719–0.894) (SVM), and that in the high-grade cancer classification was 0.835 (95% CI 0.753–0.916) (SVM). Figure 2 shows the ROC curves for the high-grade cancer classification of clinical data without data derived from ultrasound images and with that derived from ultrasound images (integrated data). The AUC of the integrated data was significantly higher than that of the clinical data (0.691 [95% CI 0.582–0.801]) in high-grade cancer classification (*p* = 0.007).

### Classification of prostate cancers in cases of systematic biopsy (n = 532)

**Figure 3:**
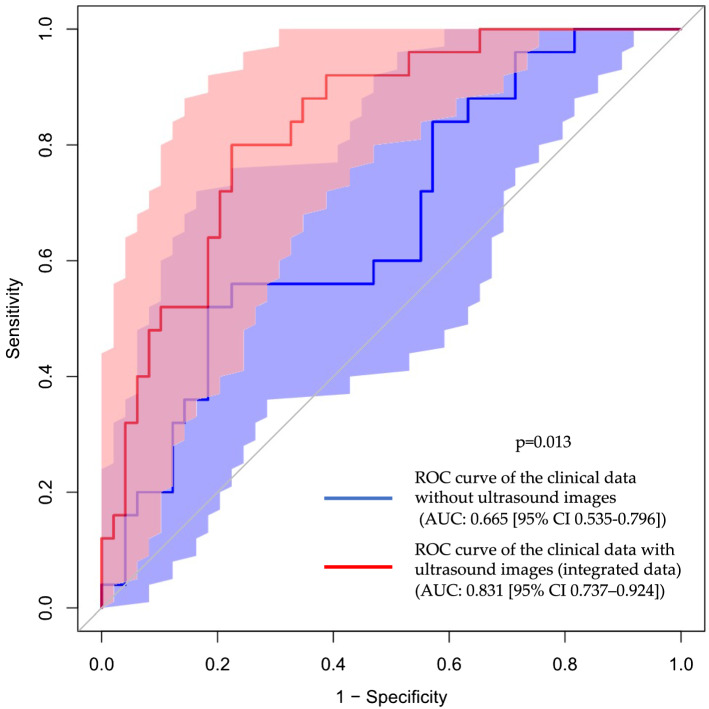
ROC curves of high-grade cancer classification (n = 532: only systematic biopsy cases). Blue line: ROC curve of the clinical data without ultrasound images. Red line: ROC curve of clinical data with ultrasound images (integrated data). Light blue area: 95% CI for the ROC curve of the clinical data without ultrasound images. Light red area: 95% CI for ROC curve of clinical data with ultrasound images (integrated data). *ROC* receiver operating characteristic, *AUC* area under the curve, *CI* confidence interval.

To eliminate selection bias based on MRI, we selected 532 cases of systematic biopsy without MRI-targeted biopsy. In image-level classification, the AUC of the cancer classification was 0.670 (95% CI 0.607–0.733), and that of the high-grade cancer classification was 0.732 (95% CI 0.658–0.807). We also applied logistic regression and SVM^24,25^ analyses based on the clinical data. The AUC of the cancer classification was 0.639 (95% CI 0.511–0.766) (SVM), and that of the high-grade cancer classification SVM was 0.665 (95% CI 0.535–0.796). Next, we applied logistic regression and SVM anlyses to three ultrasound image data pre-analyzed by deep learning. The AUC of the cancer classification was 0.722 (95% CI 0.602–0.841) (SVM), and that of the high-grade cancer classification was 0.814 (95% CI 0.708–0.920) (SVM). Finally, logistic regression and SVM anlyses were applied to the integrated data. The AUC of the cancer classification was 0.750 (95% CI 0.636–0.863) (SVM), and that of the high-grade cancer classification was 0.831 (95% CI 0.738–0.924) (SVM) (Supplementary Table S1). Supplementary Figs. S5, S6, S7, and S8 show the ROC curves for the classification accuracies. Figure 3 shows the ROC curves for the high-grade cancer classification of clinical data without data derived from ultrasound images and those derived from ultrasound images (*p* = 0.013). In AUCs, a tendency similar to that in “Classification of prostate cancers (n = 691)” was observed.

### Prostate ultrasound images of top five cases corresponding to histological cancer grading

**Figure 4:**
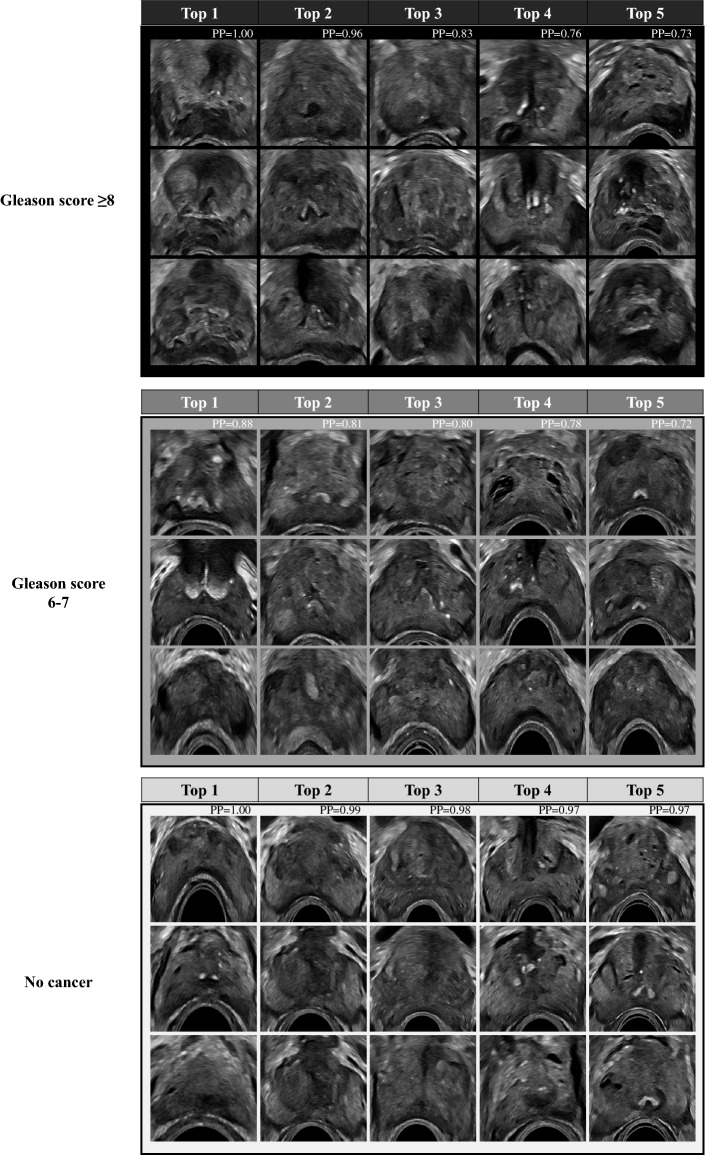
Prostate ultrasound images of top 5 cases corresponding to pathological cancer grading. Upper images: high-grade cancer cases. Middle images: low-grade cancer cases. Lower images: non-cancer cases. PP: normalized predicted probability.

Figure 4 shows the prostate ultrasound images of the top five cases according to pathological cancer grading based on the predicted probabilities of each class. The upper images show ultrasound images of high-grade cancer cases using our proposed method. The middle section shows images of the low-grade cancer cases, and the lower images show non-cancer cases. Expert urologists analyzed these images as follows. Ultrasound images of high-grade prostate cancer (upper images) show an asymmetric prostatic lobe, an unclear boundary of the prostatic capsule, and extensive hypoechoic areas. In contrast, ultrasound images of low-grade cancer (middle images) show an intensive hypoechoic area, while maintaining the clear boundary of the prostatic capsule and symmetric prostatic lobe. On the other hand, ultrasound images of non-cancer cases (lower images) show a symmetric prostatic lobe and an isoechoic area with a clear boundary of the prostatic capsule.

### A representative case with saliency map using explainable deep learning model

Supplemental Fig. S9 shows a representative case with saliency map using explainable deep learning model. We applied gradient-weighted class activation mapping (Grad-CAM) to construct a saliency map^27,28^.

## Discussion

We aimed to idenify pathological high-grade prostate cancer using ultrasound images and limited clinical data. We evaluated the prediction accuracies of the following different datasets: ultrasound image data, clinical data, and integrated data.

Several imaging examinations are performed complementary, each with its own strengths. For example, MRI contributes to the detection of significant prostate cancers. In recent years, several studies have reported the benefits of MRI prior to systematic biopsy^29^. Pellicer-Valero et al. proposed a fully automatic system based on deep learning with prostate MRI that can show cancer segmentation and cancer grading^30^. In their results, AUC of intermediate and high grade cancer detection (Gleason grade group ≥ 3) was 0.767 (ProstateX test data^31^) and 0.840 (Valencia Oncology Institute Foundation data^30^). On the othre hand, AUC when using our method of high grade cancer detection (Gleason grade group ≥ 4) was 0.835. The ROC curve showed sensitivity of 0.909 and specificity of 0.609 at a cut off value optimized for triage. Ultrasound imaging is widely used in prostate cancer screening globally because ultrasound can be easily performed at bedside. In this study, we showed that our data-driven ultrasound approach offers an efficient tool to triage patients with high-grade prostate cancers.

The main limitation of this study was that it was conducted at a single facility. It is known that high inter-observer variability deteriorates the accuracy of ultrasound diagnosis. However, we analyzed over 2500 ultrasound images. In addition, we applied an augmentation technique and transferred learning based on ImageNet^32^. In the future, we will obtain a validation set for other facilities. Furthermore, a full three-dimensional ultrasound image analysis might be able to improve the prediction accuracy in order not to miss small cancer lesions. Although further investigation should be conducted in order to reinforce our results, we hope that our method will contribute to the accurate diagnosis of prostate cancer.

Deep learning algorithms have achieved great success in medical image analyses owing to the high affinity between neural networks and images. Integrated analysis of medical multimodal data is a key factor driving practical technology in the next stage. Even if the predictive power of each datum is insufficient, data integration can improve the predictive power by appropriate machine learning techniques. Identifying the appropriate combination of data is important for better use of the data stored in the hospital. In this study, the image selection itself was performed quantitatively. This is an important step towards more accurate and integrated medical AI. Our study expands the possibility of ultrasound imaging. Recognizing pathological high-grade cancers during ultrasound procedures improves cancer management, significantly reduces mental burden on the patient, and leads to improved quality of life.

## Materials and methods

### Study population

**Figure 5:**
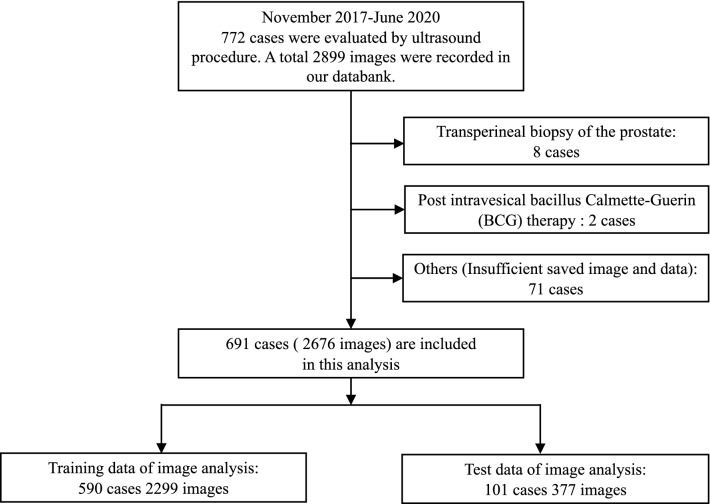
Study profile.

Our study included 772 consecutive patients and 2899 ultrasound images acquired between November 2017 and June 2020. The patients underwent ultrasound-guided prostate biopsy at Nippon Medical School Hospital in Tokyo, Japan. The study profile is shown in Fig. 5. We excluded cases with a transperineal biopsy of the prostate (eight cases), a history of post intravesical bacillus Calmette-Guerin therapy (two cases), and others (insufficient saved image and data: 71 cases). We evaluated 2676 ultrasound images (691 cases) via a transrectal approach using deep learning analysis. A systematic biopsy was performed in 532 patients. A combination of both MRI-targeted and systematic biopsies was performed in 159 cases. We divided these images into training data (November 2017 to December 2019: 590 cases, 2,299 images) and test data (January 2020–June 2020: 101 cases, 377 images). We determined the hyperparameters using only the training data (Supplementary Table S2). This study was confirmed and approved by the Institutional Review Boards of the Nippon Medical School Hospital (reference 28-11-663) and RIKEN (reference Wako3 29-14). Informed consent was obtained from all patients. All methods were carried out in accordance with relevant guidelines and regulations.

### Ultrasound and biopsy procedure

**Figure 6:**
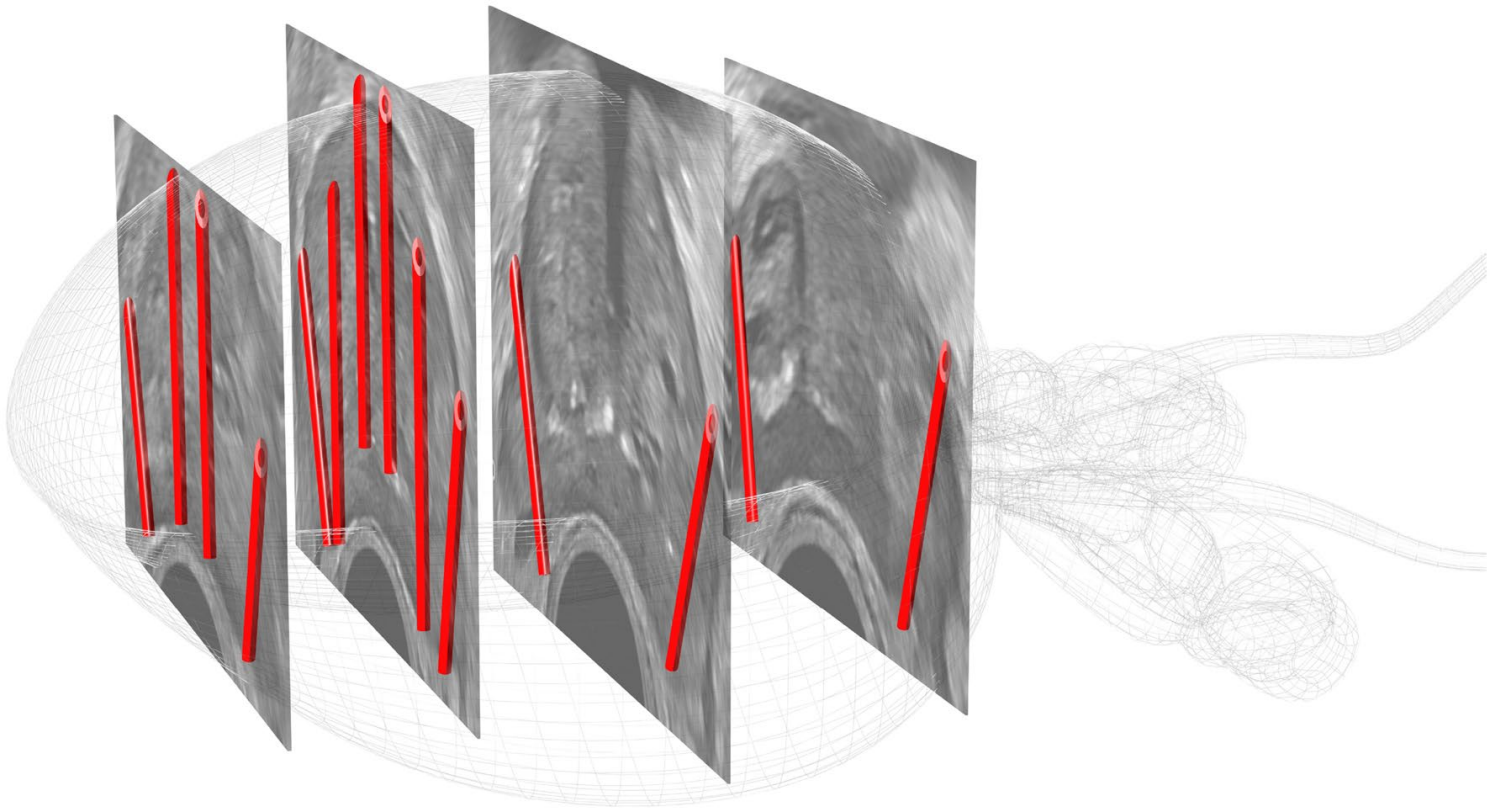
Prostate biopsy sites (systematic biopsy). Red needles indicate the prostate biopsy sites in our study.

Ultrasound images of prostate glands were saved at four locations (base, mid, apex-mid, and apex). The prostate volume was calculated for each case. Subsequently, a prostate biopsy was performed. Figure 6 shows systematic prostate biopsy sites at each location (number of biopsy sites): lateral apex (2), parasagittal apex (2), lateral apex-mid (2), parasagittal apex-mid (4), lateral mid (2), and lateral base (2). In 532 cases, a systematic biopsy was performed. Furthermore, in 159 cases, a combination of both MRI-targeted and systematic biopsies was performed. The highest biopsy Gleason score on each ultrasound image was used as the label of the corresponding images. We gave different Gleason scores for each ultrasound image. We used an ultrasound system (Aplio i800; Canon Medical Systems, Tokyo, Japan) with a 6 MHz transrectal probe (PVT-770 RT; Canon Medical System, Tokyo, Japan). All still ultrasound images were saved in Digital Imaging and Communications in Medicine (DICOM) format.

### Pathological cancer grading

Two pathologists reviewed each biopsy core and reported cancer with an assigned Gleason score. Prostate cancer was diagnosed pathologically based on the International Society of Urological Pathology grading^33^. Pathologists diagnosed all cases independently and then reached a consensus.

### Ultrasound images

All DICOM ultrasound images were converted into JPEG images. We then extracted a rectangular region of the prostate from the images. This rectangular region included proximate tissues, such as the prostatic capsular vessels, pelvic fascia, and rectum. We then adjusted these images to a size of 256 × 256 pixels. We applied an augmentation technique, including a parameter of zoom range for deep learning analysis. We provided positive or negative labels to these datasets based on the pathological grading. We defined (1) labels for the cancer group (Gleason score ≥ 6) or the other. We also defined (2) labels for the high-grade cancer group (Gleason score ≥ 8) or the other.

### Classification using a deep neural network

First, we tested three deep convolutional neural network models, Xception^23^, inceptionV3^34^, and VGG16^35^ which were pre-trained on ImageNet with classification layers adapted to our labels^32^. We selected Xception in this study because it exhibited the most precise performance for ultrasound image classification. We divided all images into test and training data based on the date of ultrasound evaluation for each case (Fig. 5). To evaluate the classification by the deep neural network, we constructed an ROC curve with the corresponding AUC. This study employed the RIKEN AIP Deep Learning Environment (RAIDEN) supercomputer system for all computations.

### Machine learning analysis for the case-level prediction

First, we applied logistic regression (ridge and lasso) and SVM anlayses only to the clinical data (age and PSA) to classify the case data. Subsequently, we applied these algorithms to three ultrasound image data pre-analyzed by deep learning (predicted probability of deep learning classification). Comparison among the different number of image sets showed that the AUC was the highest with the top three highest probabilities (|P_dl_ − 0.5|, P_dl_: predicted probability of deep learning classification) (Supplementary Table S3). We, therefore, selected ultrasound images with those three ultrasound images in every case. We used only cases with more than three images in this case-level prediction. 678 cases were remained for case-level classification. We also divided these case data into training and test sets, as described in “Study population”. Finally, we applied these algorithms to the integrated data: three ultrasound image data pre-analyzed by deep learning, TPV, PSAD, age, and PSA. We constructed an ROC curve using the corresponding AUC. We used R software for the analysis, using the glmnet package (version 2.0.16) for the ridge and lasso regression and the e1071 package (version 1.7.0) for the SVM. Calculations were performed automatically using the software packages.

### Prostate ultrasound images corresponding to histological cancer grading

We evaluated prostate ultrasound images corresponding to pathological cancer grading based on two types of labels: (1) the cancer classification label and (2) the high-grade cancer classification label. We selected the top five highest predicted probability cases from the high-grade cancer logistic regression (ridge) as representative cases corresponding to the high-grade cancer group. We selected the top five lowest predicted probability cases from the cancer logistic regression as representative cases corresponding to the non-cancer group. We defined the top five highest predicted probability cases of cancer without high-grade cancer as the low-grade cancer group.

### Saliency map

We applied Grad-CAM to construct a saliency map for deep learning analysis^27,28^. Grad-CAM is a technique used to produce visual explanations of decisions made by convolutional neural networks.

### Statistical analysis

We compared the characteristics of patients who were cancer cases or non-cancer cases using the Wilcoxon rank-sum test for continuous data. The ROC curves were constructed and compared using the ‘pROC’ (version 1.13.0) package in R^36^. All reported *p* values were two-sided, with the level of statistical significance set at* p* < 0.05.

## Supplementary Information

Supplementary Information.

## Data Availability

The clinical datasets used were collected at the Nippon Medical School Hospital. They are not publicly available, and restrictions apply to their use.
