# Supplementary material for: A data-driven ultrasound approach discriminates pathological high grade prostate cancer
Source: Sci Rep. 2022 Jan 17;12:860. doi: 10.1038/s41598-022-04951-3 (PMC8764059; doi:10.1038/s41598-022-04951-3)
Supplement: Supplementary file 1 — Supplementary Information. [file 41598_2022_4951_MOESM1_ESM.pdf]

Supplementary Table S1

	Images	Clinical data	Image integration	Data integration	*p*-value*
Cancer classification	Deep learning<br>0.670<br>[95% CI 0.607–0.733]	Ridge<br>0.633<br>(95% CI 0.505–0.761)	Deep learning + Ridge<br>0.719<br>(95% CI, 0.600–0.839)	Deep learning + Ridge<br>0.735<br>(95% CI 0.617–0.852)	0.195
		Lasso<br>0.633<br>(95% CI 0.505–0.761)	Deep learning + Lasso<br>0.718<br>(95% CI, 0.599–0.838)	Deep learning + Lasso<br>0.727<br>(95% CI 0.609–0.846)	0.255
		**SVM<br>0.639<br>(95% CI 0.511–0.766)**	**Deep learning + SVM<br>0.722<br>(95% CI, 0.602–0.841)**	**Deep learning + SVM<br>0.750<br>(95% CI 0.636–0.863)**	0.299
High-grade cancer classification	Deep learning<br>0.732<br>[95% CI 0.658–0.807]	Ridge<br>0.636<br>(95% CI 0.504–0.768)	Deep learning + Ridge<br>0.811<br>(95% CI, 0.704–0.919)	Deep learning + Ridge<br>0.814<br>(95% CI 0.715–0.913)	0.020
		Lasso<br>0.636<br>(95% CI 0.504–0.768)	Deep learning + Lasso<br>0.811<br>(95% CI, 0.704–0.919)	Deep learning + Lasso<br>0.815<br>(95% CI 0.711–0.919)	0.009
		**SVM<br>0.665<br>(95% CI 0.535–0.796)**	**Deep learning + SVM<br>0.814<br>(95% CI, 0.708–0.920)**	**Deep learning + SVM<br>0.831<br>(95% CI 0.738–0.924)**	0.013

Supplementary Table S1: AUCs of the cancer grading classification (n=532: only systematic biopsy cases)

The bold text indicates the highest value of AUCs. * AUC of clinical data versus that of data integration

AUC: area under the curve; CI: confidence Interval; SVM: support vector machine

Supplementary Table S2

**Hyper parameters of the deep learning algorithms for image classification**
The epoch was set to 20 and batch size was set to 4. Learning rate was 0.001.
Keras's ImageDataGenerator was used for image augmentation using the following parameters:
featurewise_center=False, samplewise_center=False, featurewise_std_normalization=False, samplewise_std_normalization=False, zca_whitening=False, rotation_range=0, width_shift_range=0.01, height_shift_range=0.01, shear_range=0., zoom_range=0.01, channel_shift_range=0., fill_mode='nearest', cval=0., horizontal_flip=True, vertical_flip=False, rescale=1. / 255.

Supplementary Table S2: **Hyper parameters of a deep learning algorithm for image classification**

Supplementary Table S3

	top1	top2	top3	top4
Ridge	0.785 (95% CI 0.690–0.879)	0.821 (95% CI 0.737–0.906)	0.822 (95% CI 0.736–0.908)	0.812 (95% CI 0.723–0.901)
sensitivity	0.424	0.788	0.879	0.727
specificity	0.969	0.688	0.625	0.797
Lasso	0.786 (95% CI 0.692–0.880)	0.826 (95% CI 0.743–0.909)	0.824 (95% CI 0.737–0.911)	0.809 (95% CI 0.719–0.899)
sensitivity	0.697	0.818	0.848	0.758
specificity	0.703	0.672	0.703	0.781
SVM	0.790 (95% CI 0.697–0.883)	0.834 (95% CI 0.753–0.915)	**0.835 (95% CI 0.753–0.916)**	0.814 (95% CI 0.726–0.903)
sensitivity	0.697	0.848	0.788	0.758
specificity	0.719	0.703	0.750	0.797

Supplementary Table S3: AUC comparison among different image sets.

The bold text indicates the highest value of AUCs.

AUC: area under the curve; CI: confidence Interval; SVM: support vector machine

Supplementary Figure S1

Cancer classification

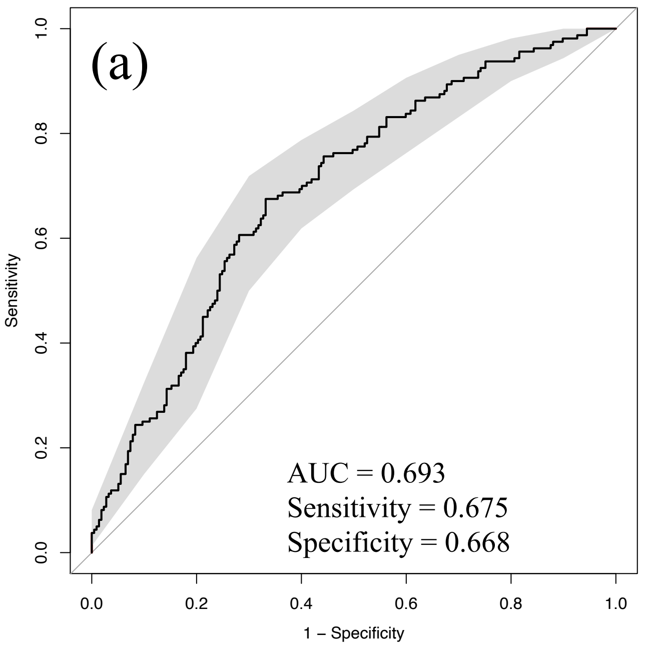

High-grade cancer classification

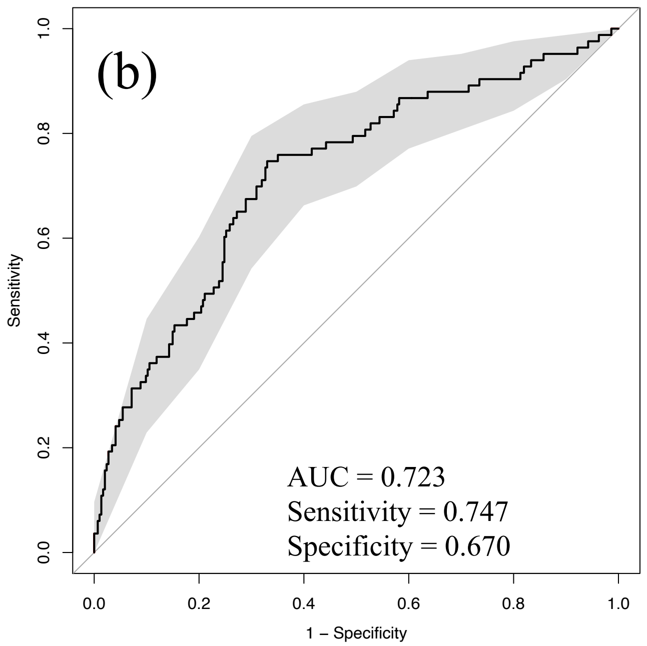

Supplementary Figure S1: ROC curves for classification on ultrasound images

(a): Cancer classification; (b): High-grade cancer classification, The grey area represents the 95% CI. ROC: receiver operating characteristic, CI: confidence interval

We found the cut-off value using the Youden index.

Supplementary Figure S2

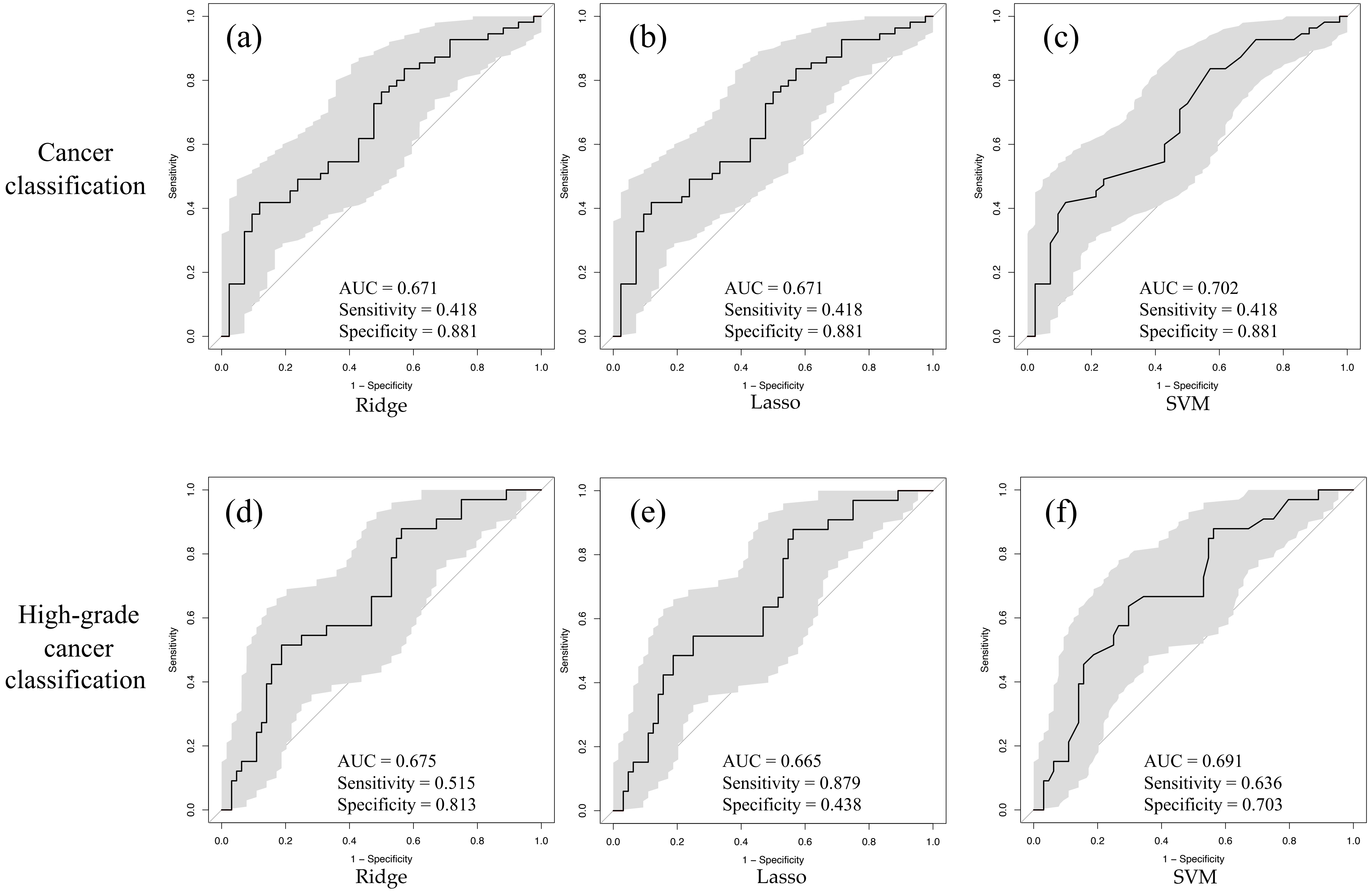

Supplementary Figure S2: **ROC curves of case-level classifications based on clinical data**

(a): Cancer classification (Ridge); (b): Cancer classification (Lasso); (c): Cancer classification (SVM); (d): High-grade cancer classification (Ridge); (e): High-grade cancer classification (Lasso); (f): High-grade cancer classification (SVM), The grey area represents the 95% CI. ROC: receiver operating characteristic; SVM: support vector machine; CI: confidence interval.

We found the cut-off value using the Youden index.

Supplementary Figure S3

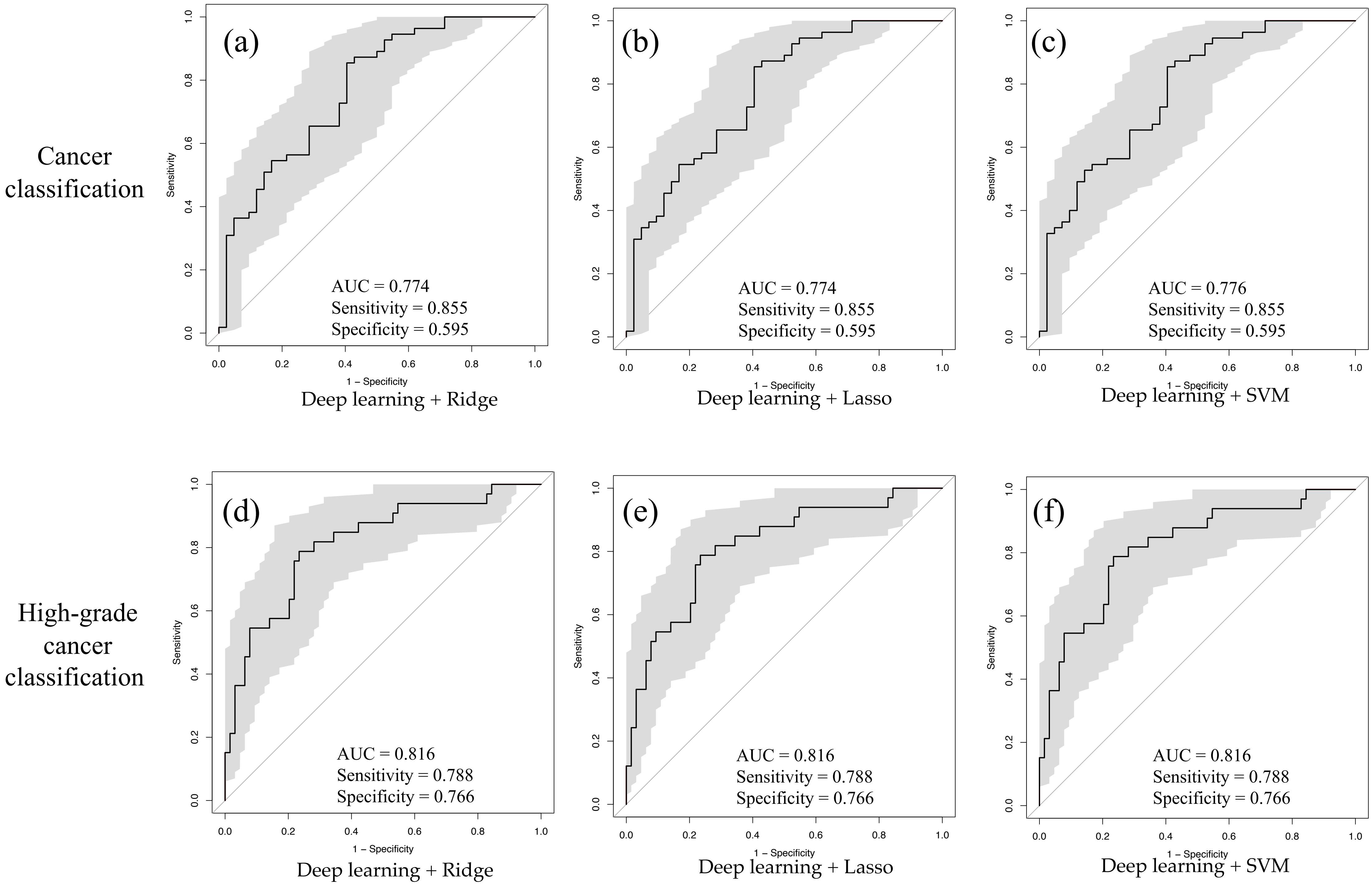

Supplementary Figure S3: **ROC curves of Case-level classification using an image integration approach**

(a): Cancer classification (Deep learning + Ridge); (b): Cancer classification (Deep learning + Lasso); (c): Cancer classification (Deep learning + SVM); (d): High-grade cancer classification (Deep learning + Ridge); (e): High-grade cancer classification (Deep learning + Lasso); (f): High-grade cancer classification (Deep learning + SVM)

The grey area represents the 95% CI. ROC: receiver operating characteristic; SVM: support vector machine; CI: confidence interval. We found the cut-off value using the Youden index

Supplementary Figure S4

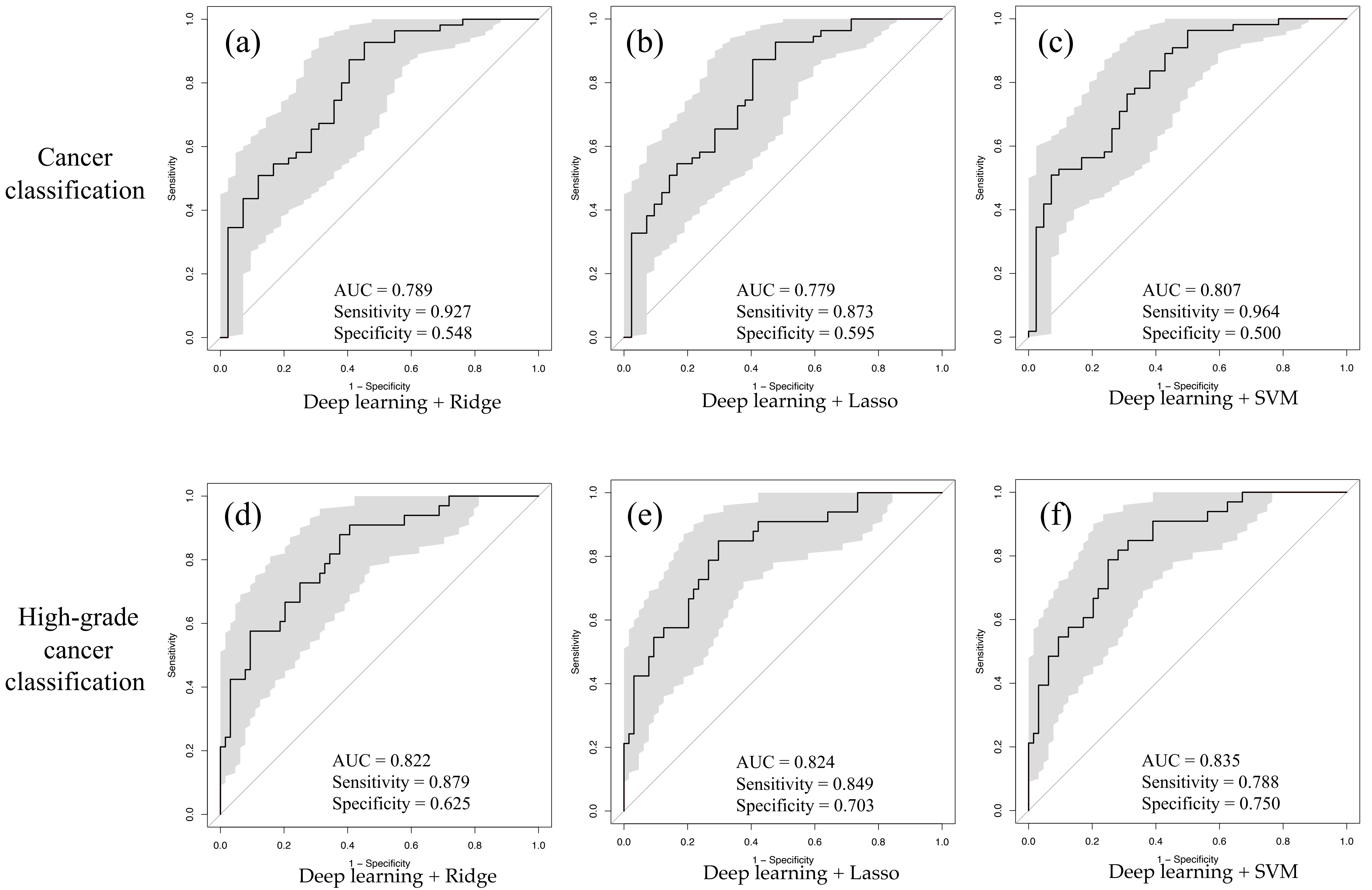

Supplementary Figure S4: **ROC curves of Case-level classification using a data integration approach**

(a): Cancer classification (Deep learning + Ridge); (b): Cancer classification (Deep learning + Lasso); (c): Cancer classification (Deep learning + SVM); (d): High-grade cancer classification (Deep learning + Ridge); (e): High-grade cancer classification (Deep learning + Lasso); (f): High-grade cancer classification (Deep learning + SVM)

The grey area represents the 95% CI. ROC: receiver operating characteristic; SVM: support vector machine; CI: confidence interval. We found the cut-off value using the Youden index.

Supplementary Figure S5

Cancer classification

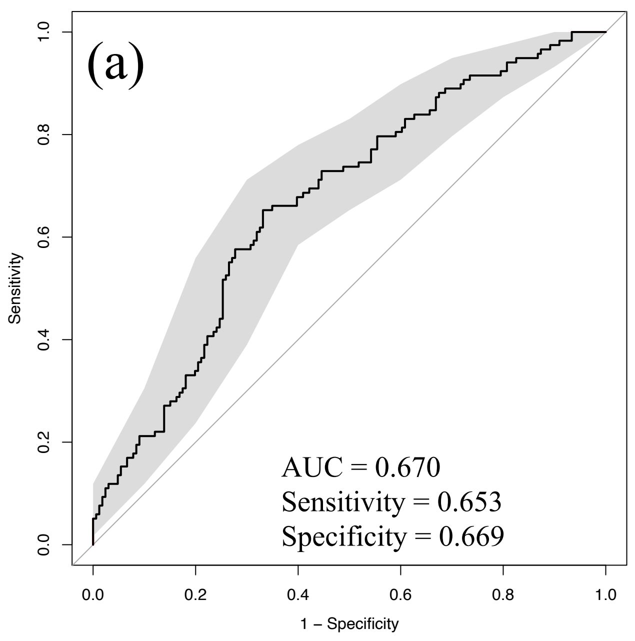

High-grade cancer classification

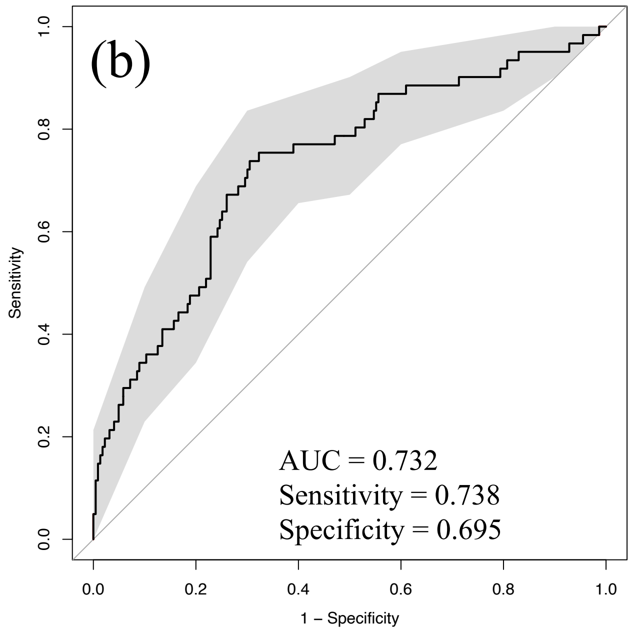

Supplementary Figure S5: **ROC curves for classification on ultrasound images (n=532: only systematic biopsy cases)**

(a): Cancer classification; (b): High-grade cancer classification The grey area represents the 95% CI. ROC: receiver operating characteristic, CI: confidence interval

We found the cut-off value using the Youden index

Supplementary Figure S6

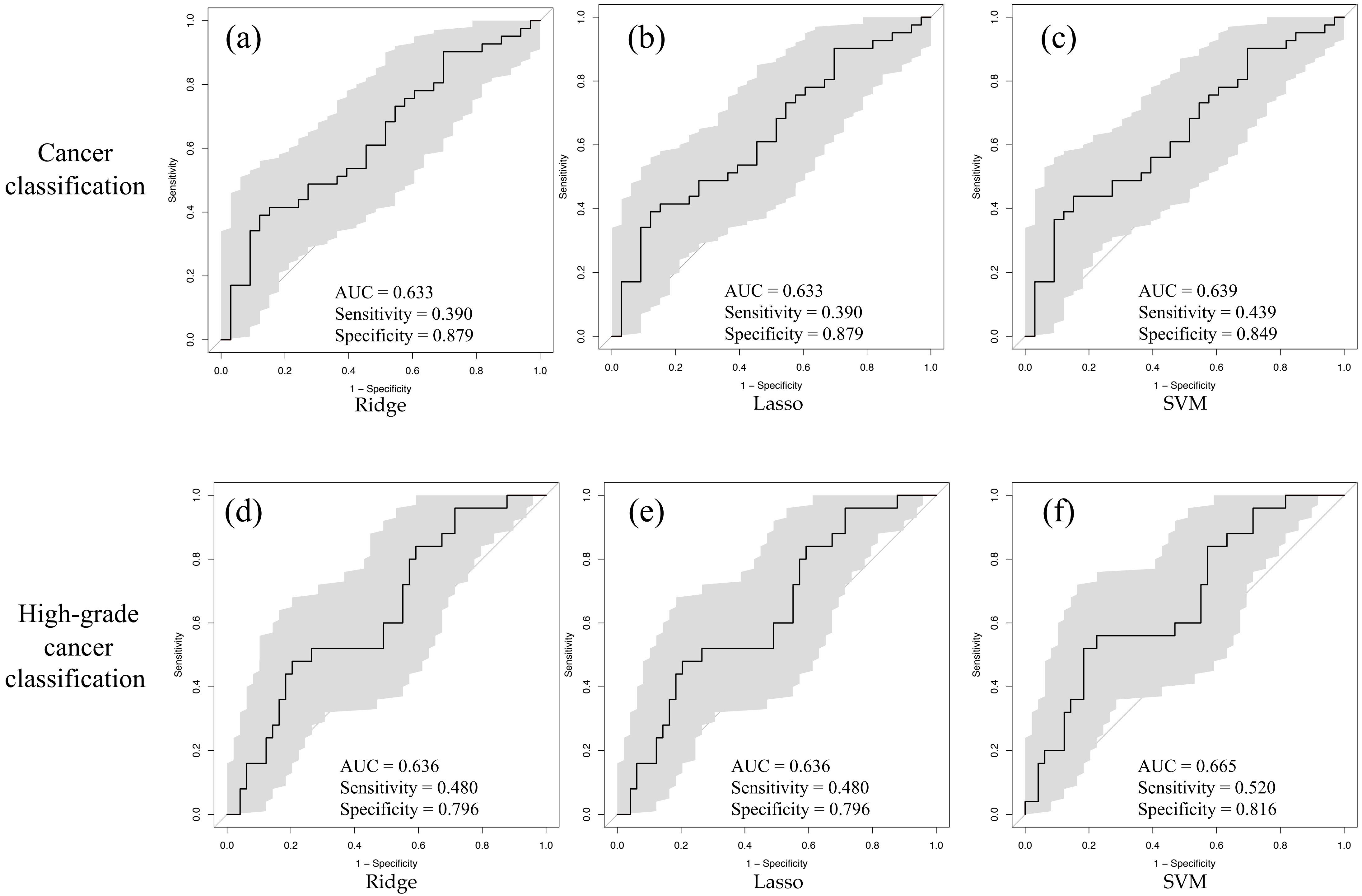

Supplementary Figure S6: **ROC curves of case-level classifications based on clinical data (n=532: only systematic biopsy cases)**

(a): Cancer classification (Ridge); (b): Cancer classification (Lasso); (c): Cancer classification (SVM); (d): High-grade cancer classification (Ridge); (e): High-grade cancer classification (Lasso); (f): High-grade cancer classification (SVM), The grey area represents the 95% CI. ROC: receiver operating characteristic; SVM: support vector machine; CI: confidence interval. We found the cut-off value using the Youden index.

Supplementary Figure S7

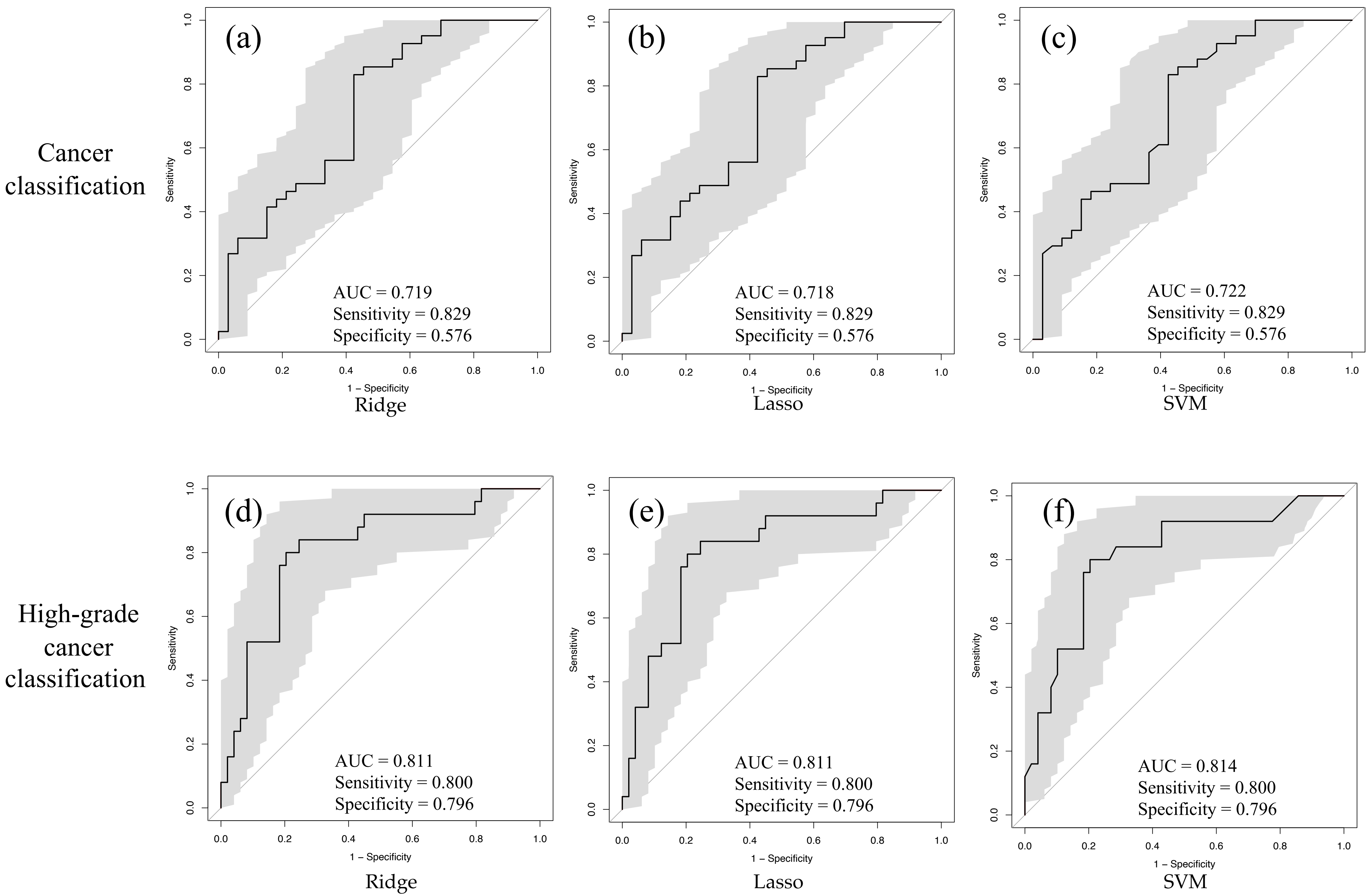

Supplementary Figure S7: **ROC curves of Case-level classification using an image integration approach (n=532: only systematic biopsy cases)**

(a): Cancer classification (Deep learning + Ridge); (b): Cancer classification (Deep learning + Lasso); (c): Cancer classification (Deep learning + SVM); (d): High-grade cancer classification (Deep learning + Ridge); (e): High-grade cancer classification (Deep learning + Lasso); (f): High-grade cancer classification (Deep learning + SVM); The grey area represents the 95% CI. ROC: receiver operating characteristic; SVM: support vector machine; CI: confidence interval. We found the cut-off value using the Youden index.

Supplementary Figure S8

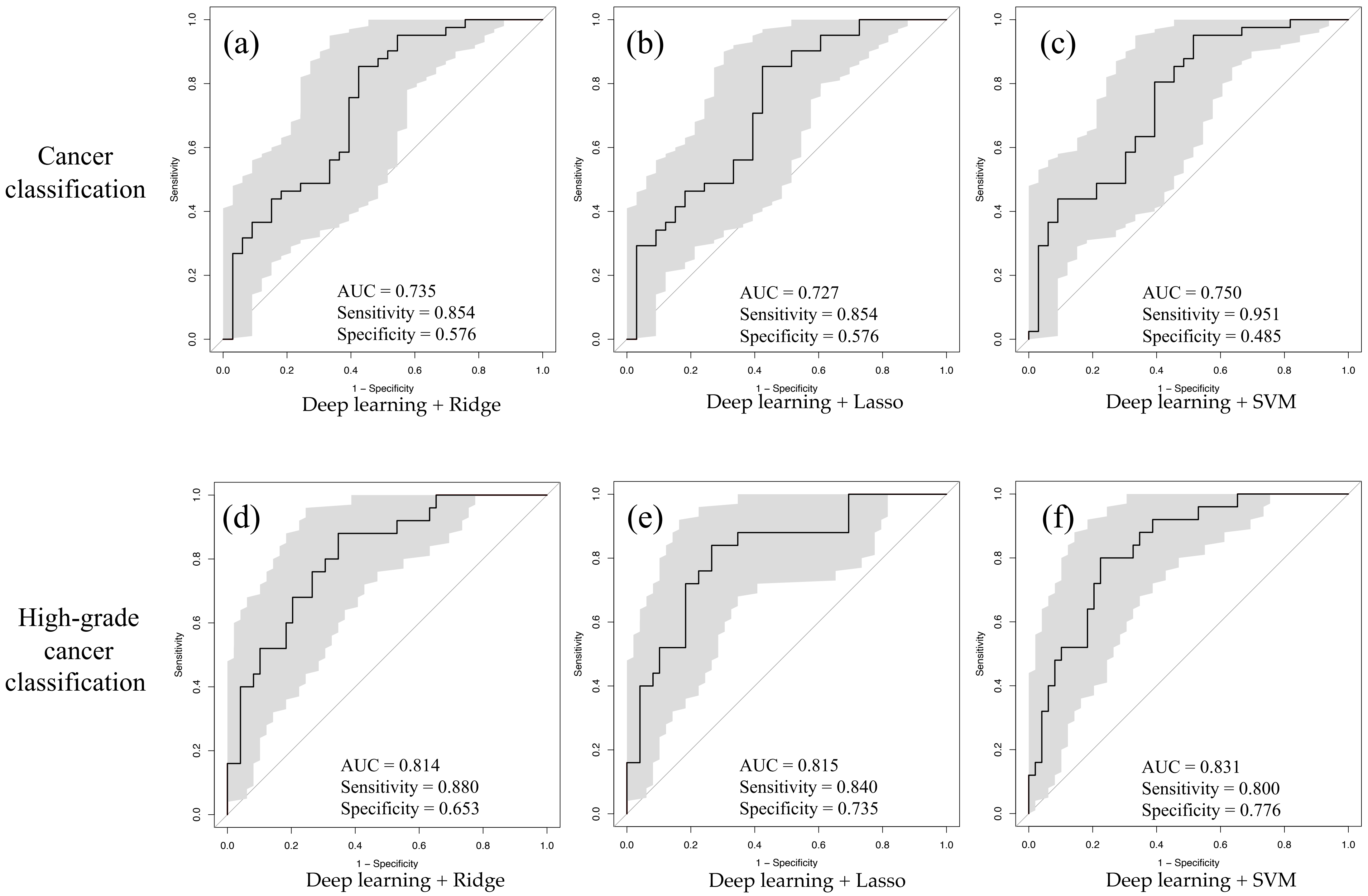

Supplementary Figure S8: **ROC curves of ROC curves of Case-level classification using a data integration approach (n=532: only systematic biopsy cases)**

(a): Cancer classification (Deep learning + Ridge); (b): Cancer classification (Deep learning + Lasso); (c): Cancer classification (Deep learning + SVM); (d): High-grade cancer classification (Deep learning + Ridge); (e): High-grade cancer classification (Deep learning + Lasso); (f): High-grade cancer classification (Deep learning + SVM)

The grey area represents the 95% CI. ROC: receiver operating characteristic; SVM: support vector machine; CI: confidence interval. We found the cut-off value using the Youden index.

Supplementary Figure S9

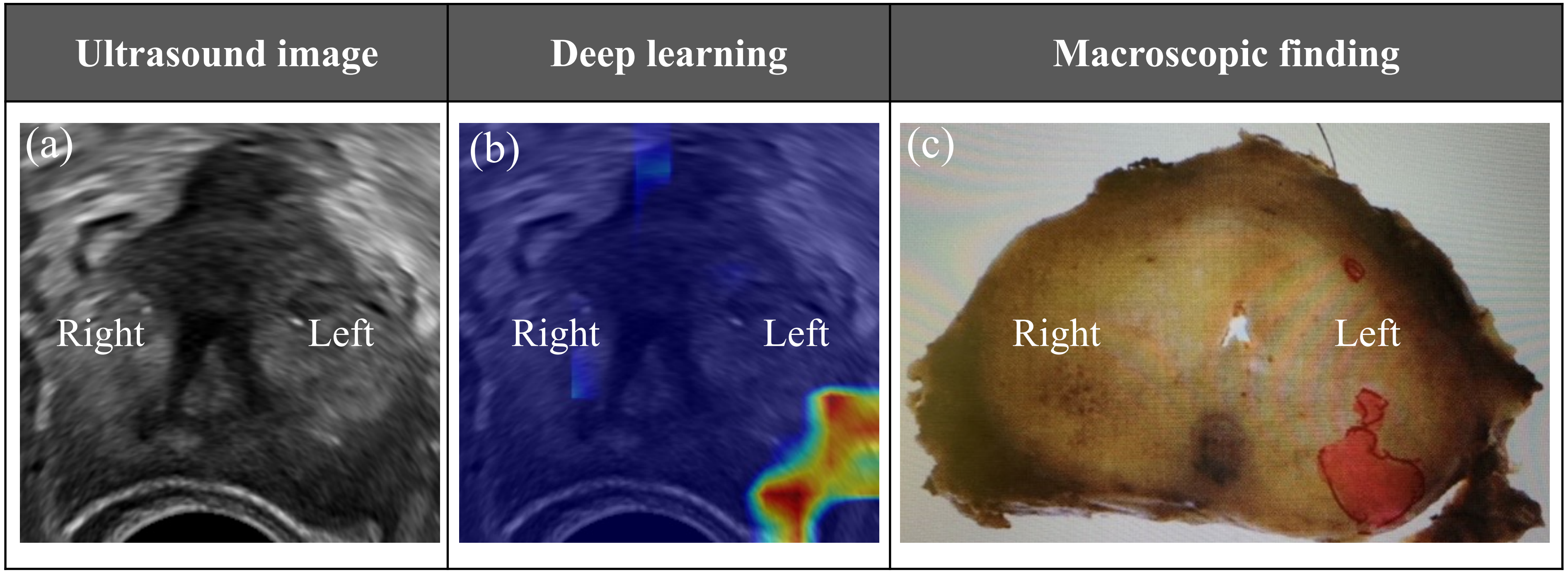

Supplementary Figure S9: Representative case with a saliency map using explainable deep learning model
(a): gray-scale ultrasound image of prostate gland; (b): saliency map using an explainable deep learning model on an ultrasound image; (c): macroscopic result (red area indicates cancer location).

A 62-y-old man exhibited a PSA level of 5.7 ng/mL in a routine evaluation. A digital rectal examination revealed a slightly enlarged prostate. A prostatic biopsy revealed Gleason score of 8 (4+4) adenocarcinoma in the left peripheral zone. He underwent a robot-assisted laparoscopic radical prostatectomy, and the pathology revealed a Gleason score of 8 (4+4) adenocarcinoma. The left image (a) is a gray-scale ultrasound image of the prostate gland. The middle image (b) is a saliency map using an explainable deep learning model on an ultrasound image. The red area of the saliency map mathematically indicates a deep-learning-focused region. The right image (c) shows a macroscopic image.
